# Supplementary figures and images for: Integrating the RFID identification system for Charolaise breeding bulls with 3D imaging for virtual archive creation
Source: PeerJ Comput Sci. 2019 Mar 4;5:e179. doi: 10.7717/peerj-cs.179 (PMC7924494; doi:10.7717/peerj-cs.179)

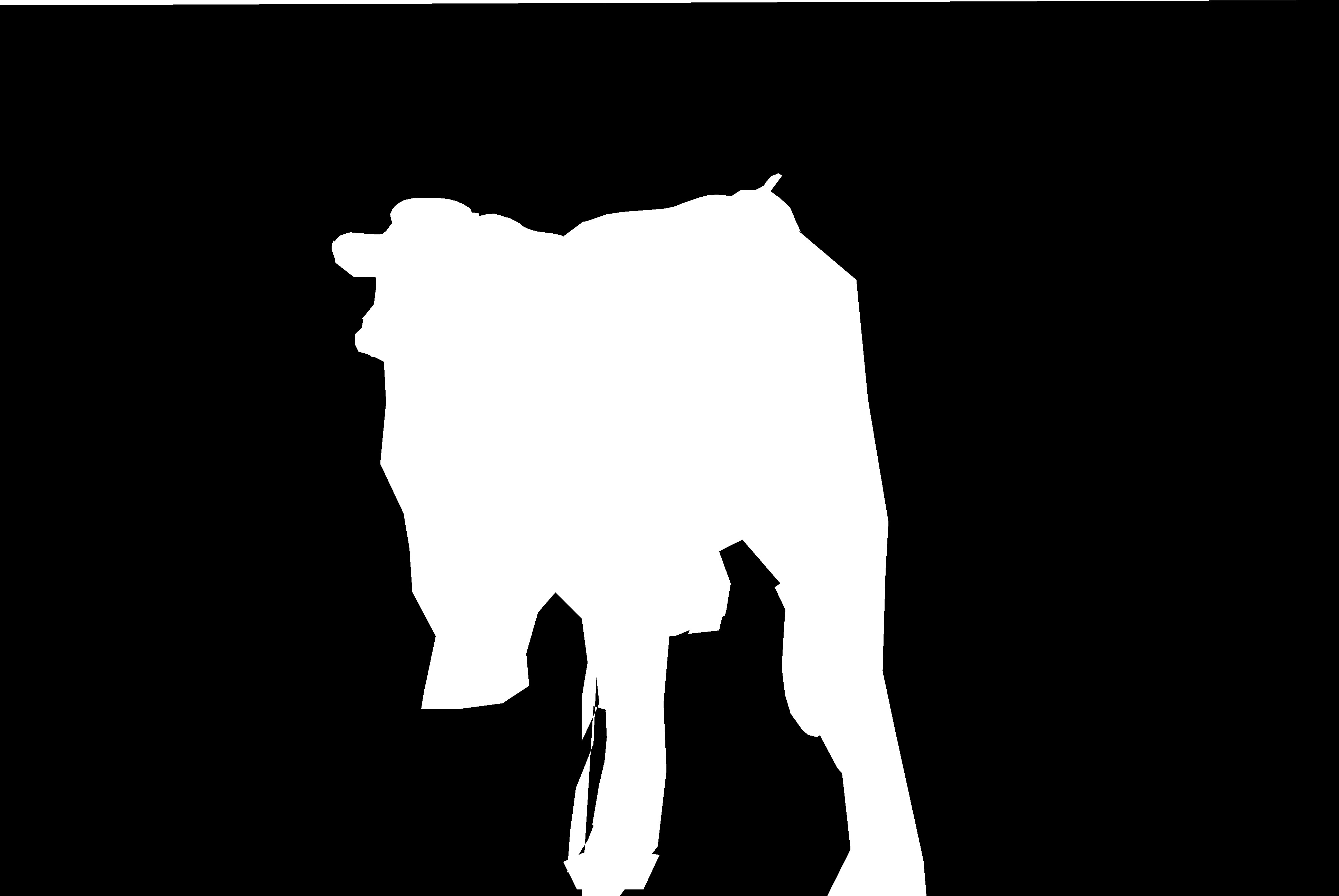

Supplement: Supplemental Information 3 — Different masks of the bull during model creation. [file peerj-cs-05-179-s003.zip › MASK per toro/6105 toro.jpg]

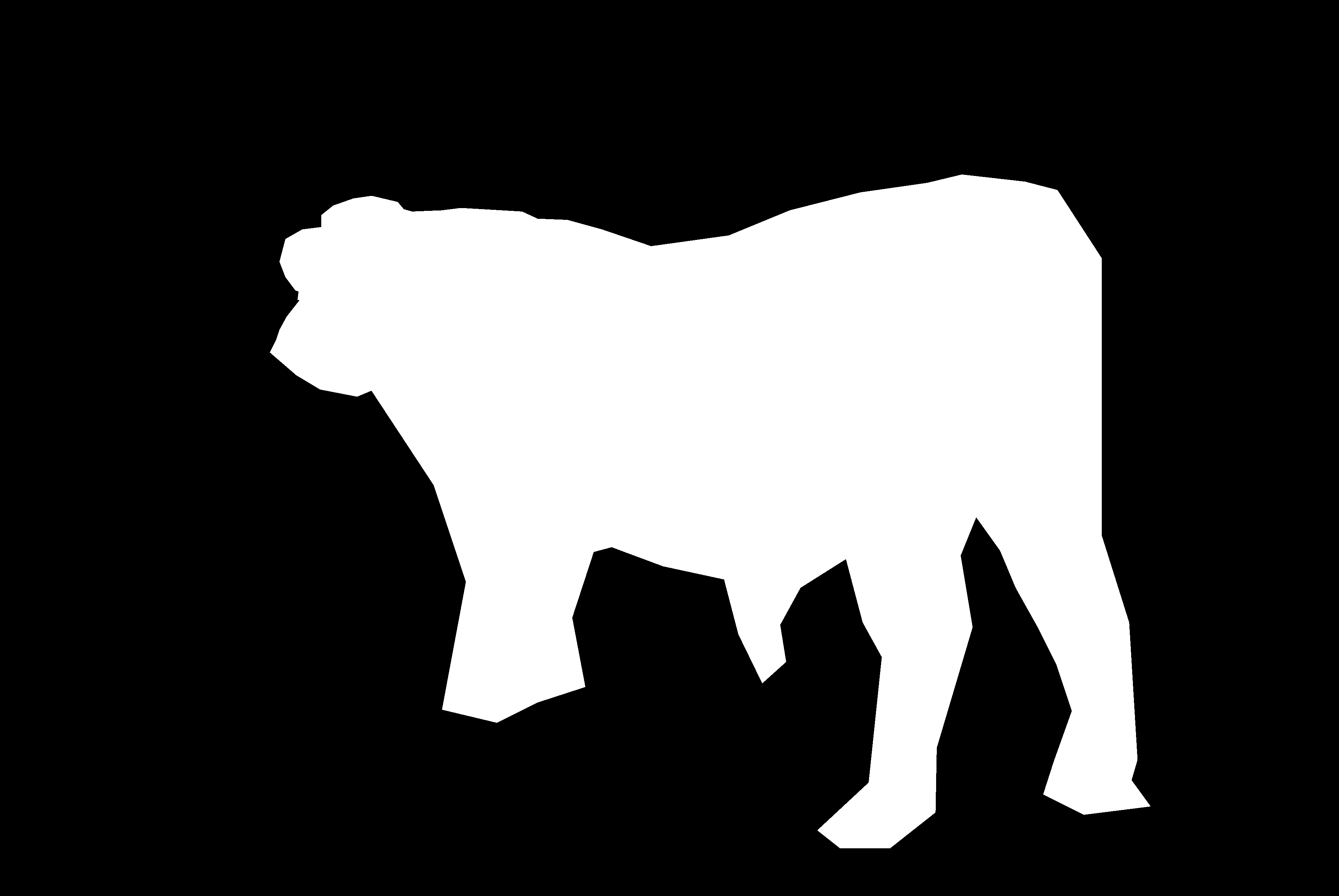

Supplement: Supplemental Information 3 — Different masks of the bull during model creation. [file peerj-cs-05-179-s003.zip › MASK per toro/DSC 6125.png]

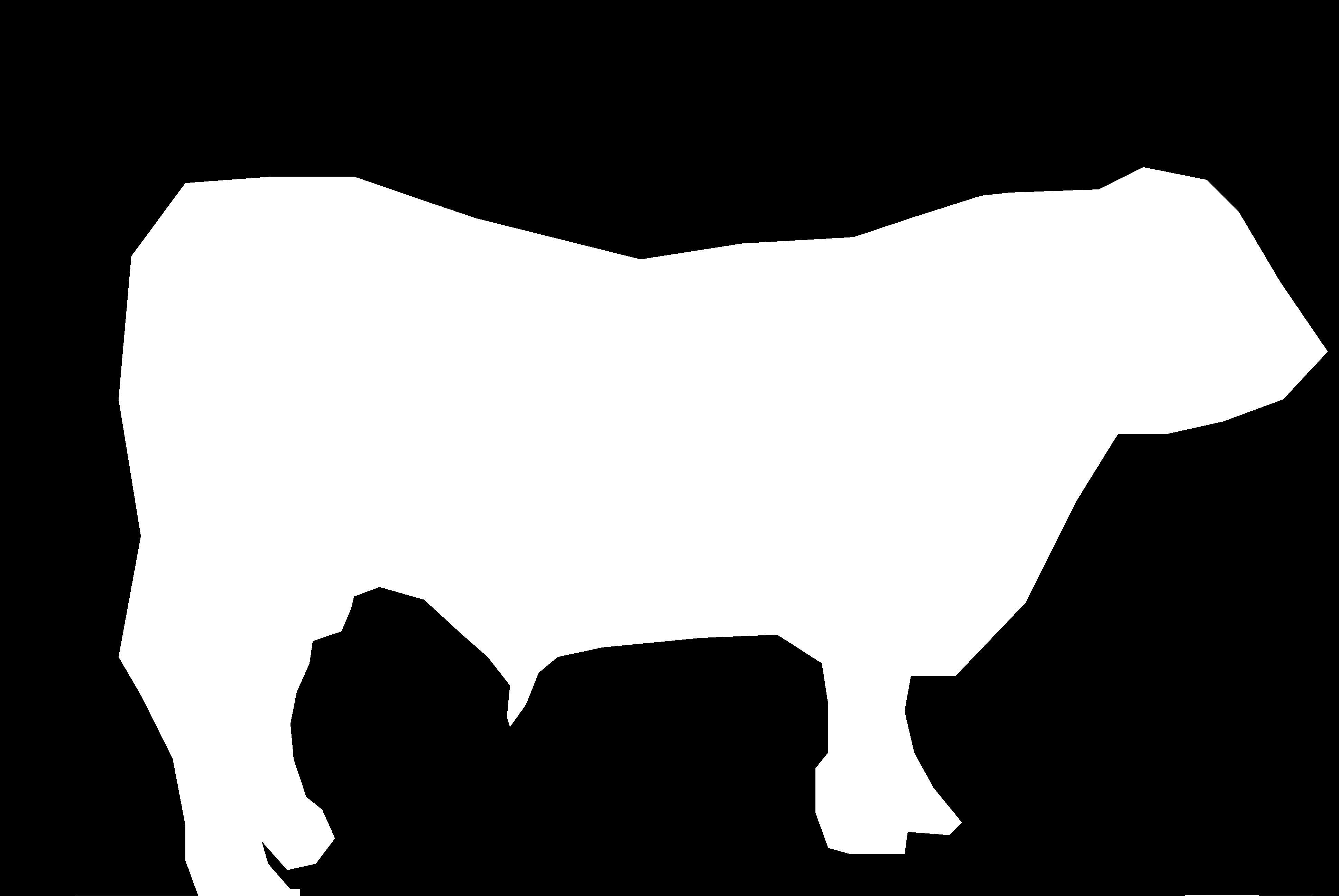

Supplement: Supplemental Information 3 — Different masks of the bull during model creation. [file peerj-cs-05-179-s003.zip › MASK per toro/DSC6066.png]

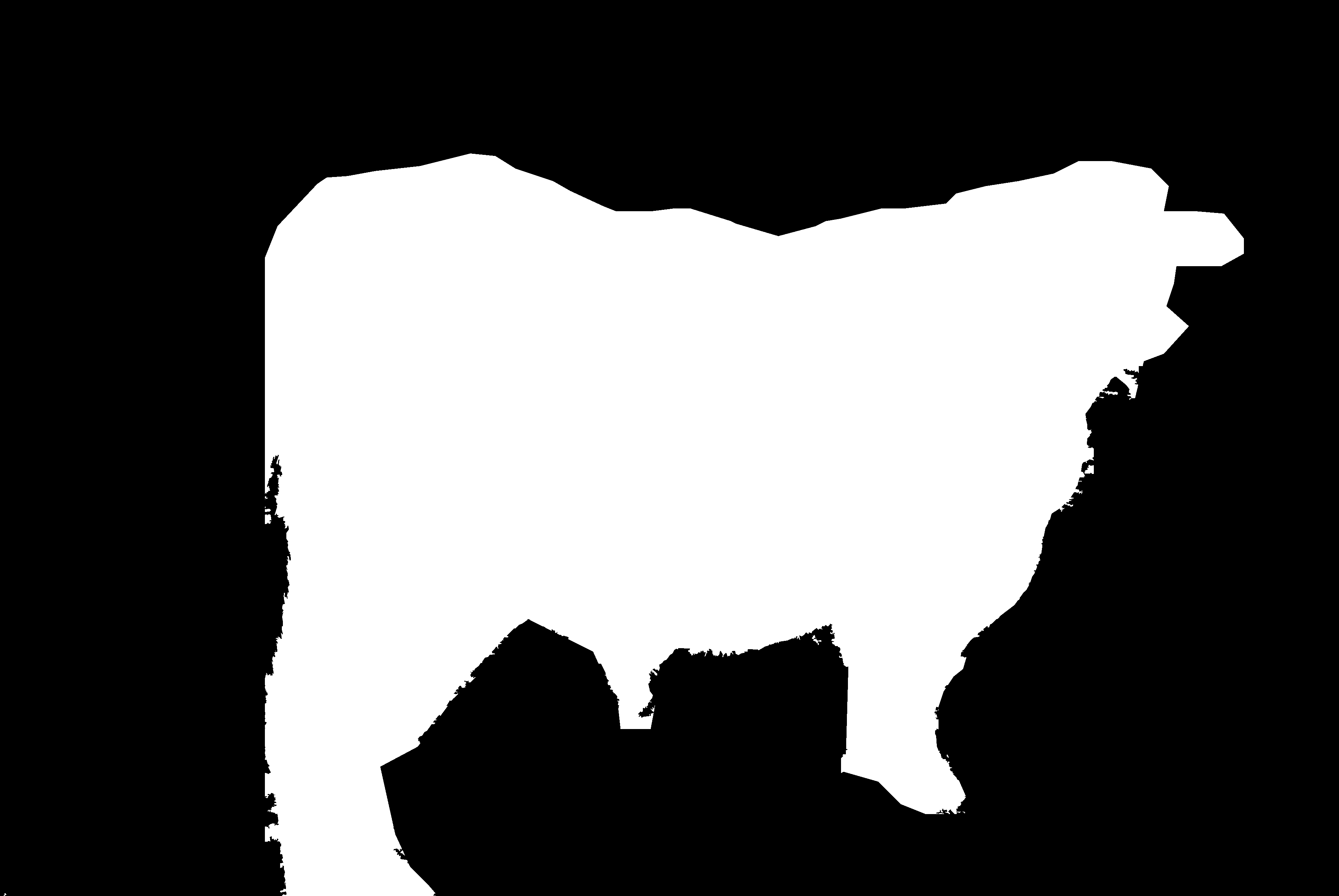

Supplement: Supplemental Information 3 — Different masks of the bull during model creation. [file peerj-cs-05-179-s003.zip › MASK per toro/DSC6075.png]

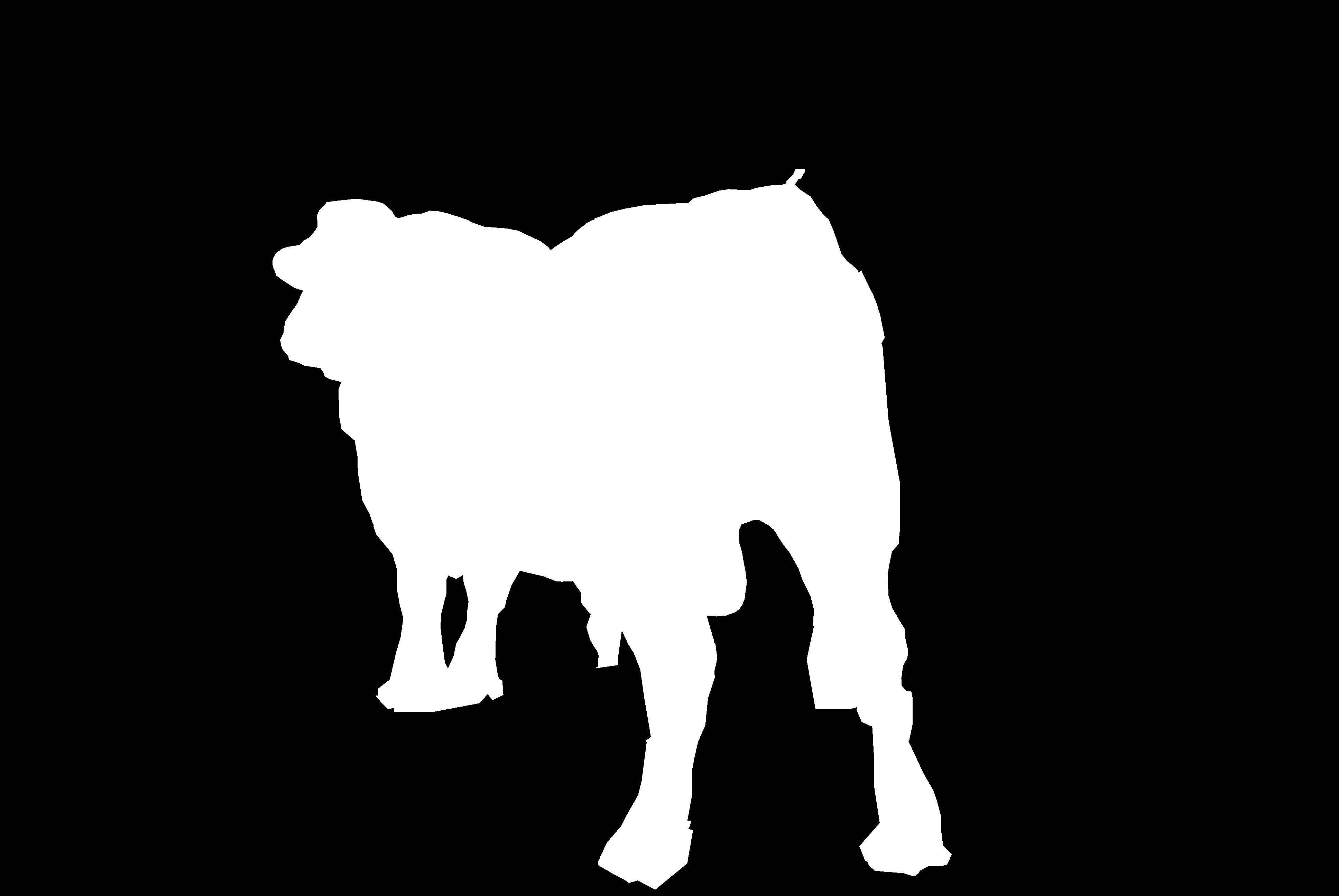

Supplement: Supplemental Information 3 — Different masks of the bull during model creation. [file peerj-cs-05-179-s003.zip › MASK per toro/DSC6112.PNG]

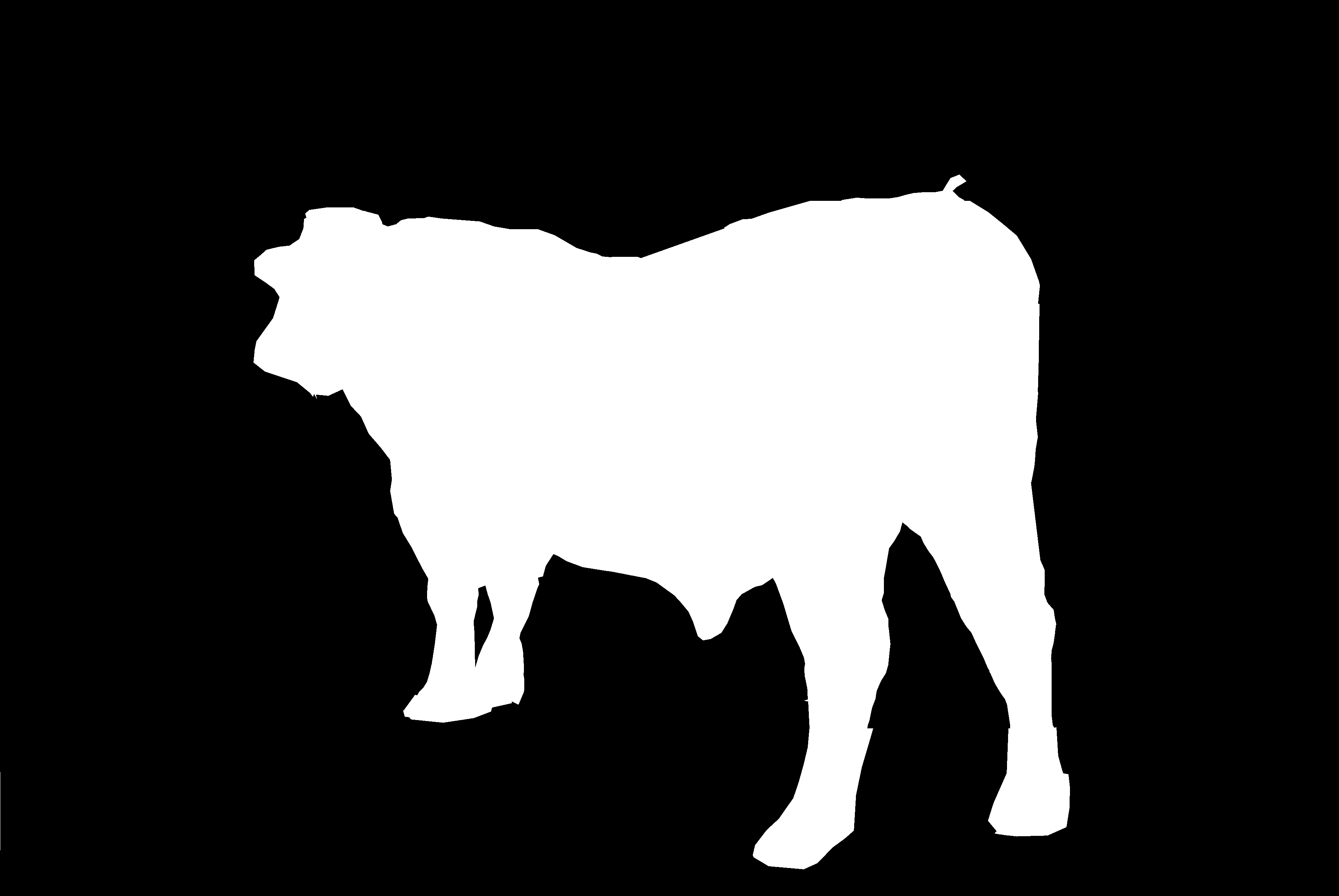

Supplement: Supplemental Information 3 — Different masks of the bull during model creation. [file peerj-cs-05-179-s003.zip › MASK per toro/dsc6121.png]

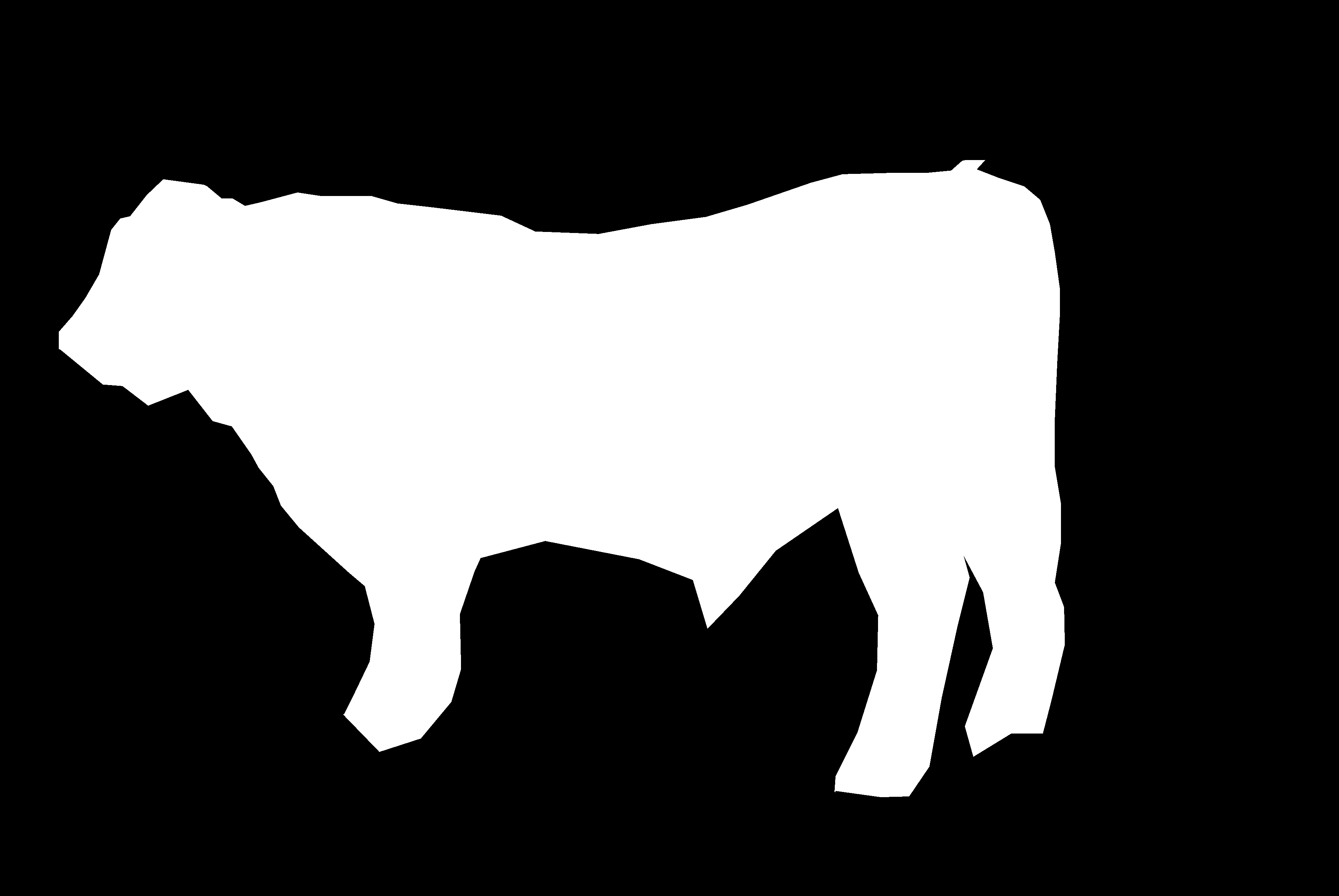

Supplement: Supplemental Information 3 — Different masks of the bull during model creation. [file peerj-cs-05-179-s003.zip › MASK per toro/DSC6135.png]

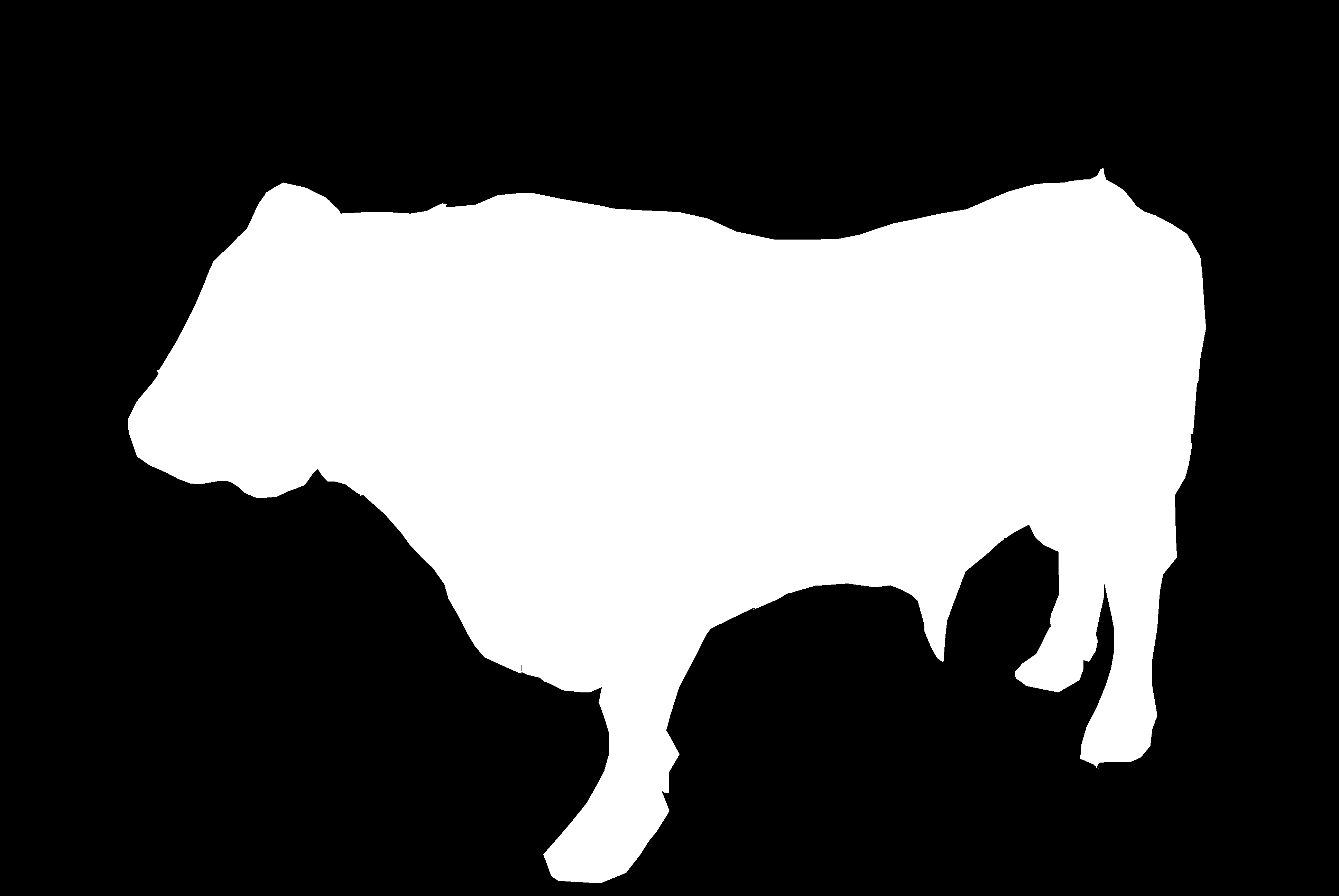

Supplement: Supplemental Information 3 — Different masks of the bull during model creation. [file peerj-cs-05-179-s003.zip › MASK per toro/DSC6136.png]

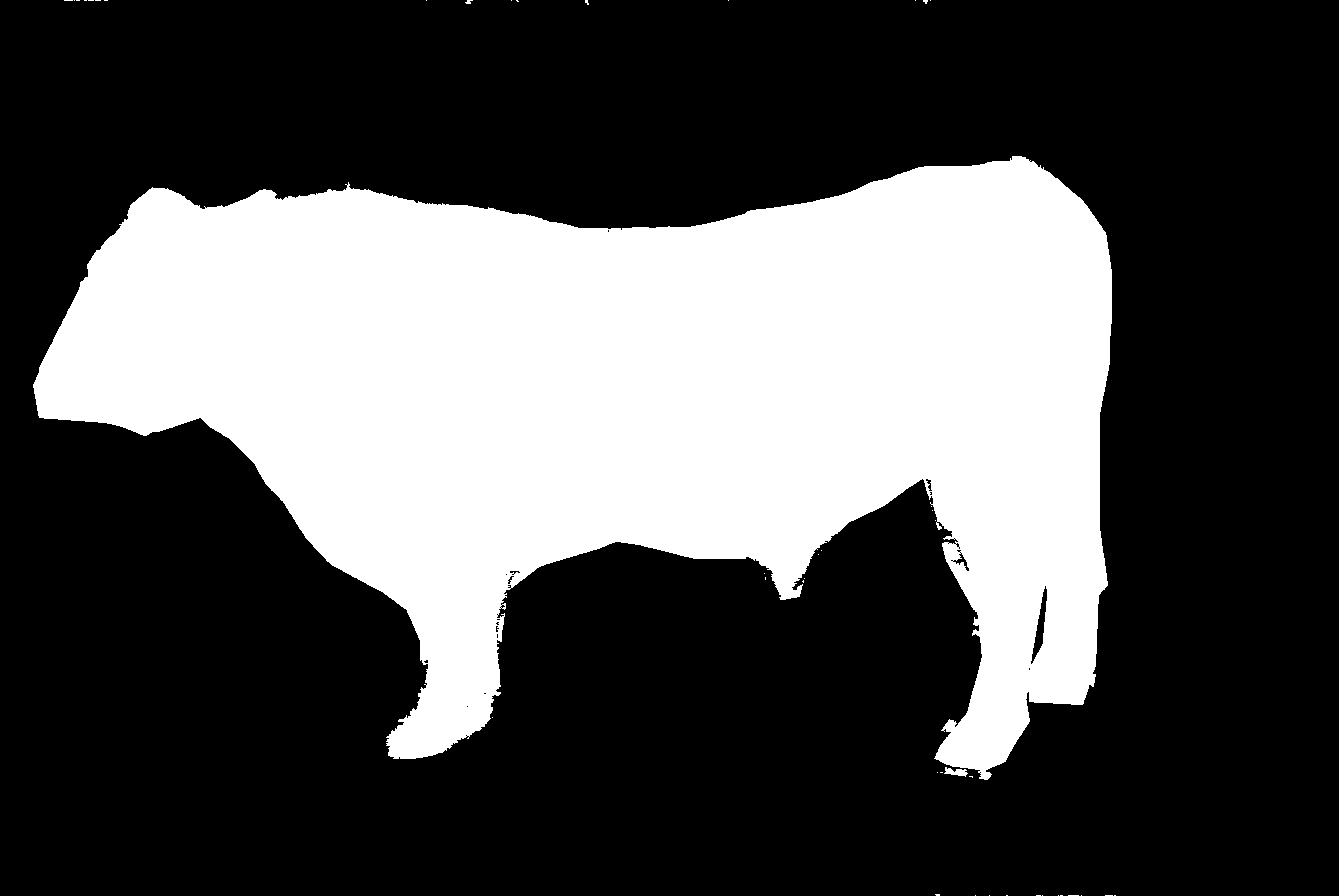

Supplement: Supplemental Information 3 — Different masks of the bull during model creation. [file peerj-cs-05-179-s003.zip › MASK per toro/DSC6140.png]

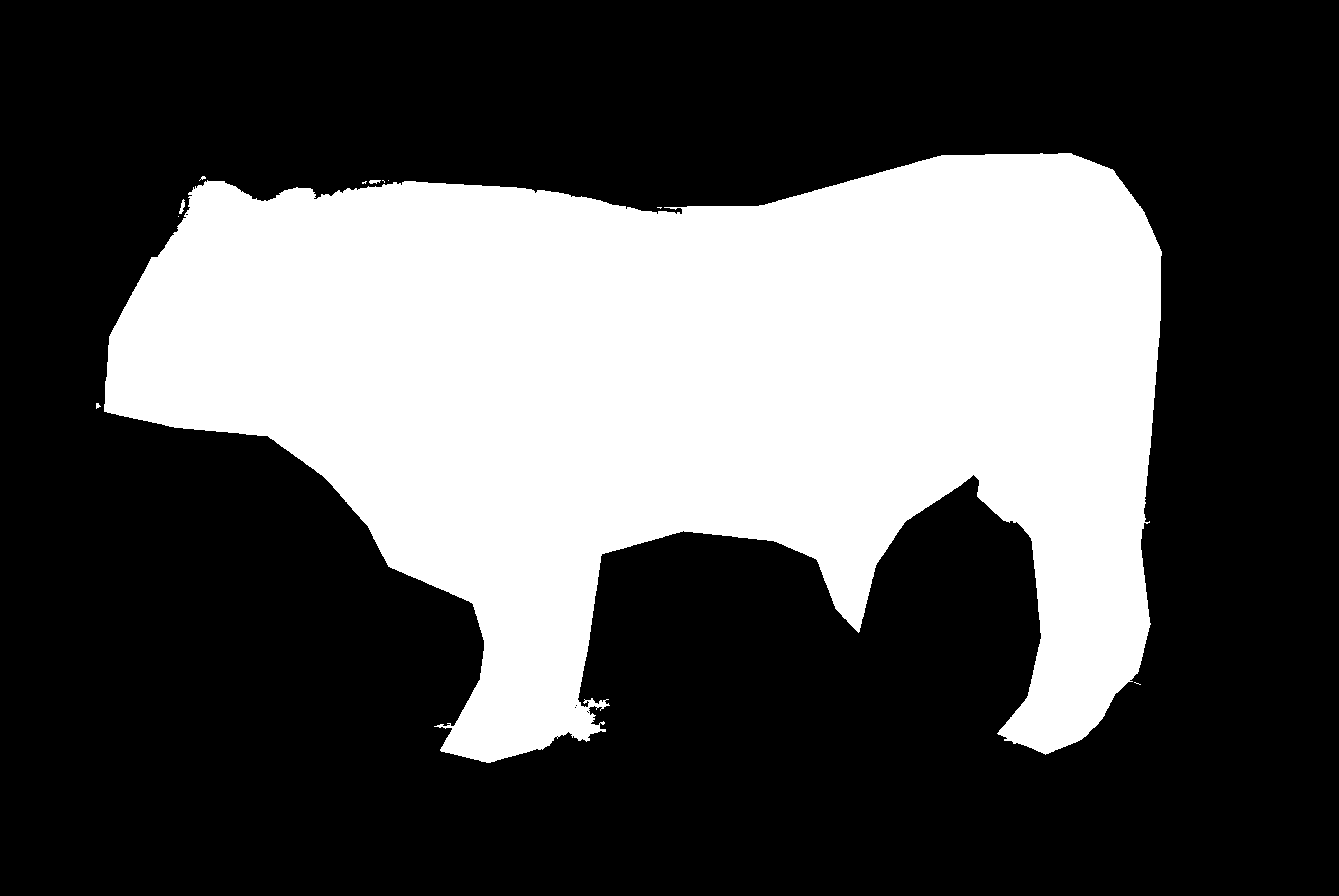

Supplement: Supplemental Information 3 — Different masks of the bull during model creation. [file peerj-cs-05-179-s003.zip › MASK per toro/DSC6144.png]

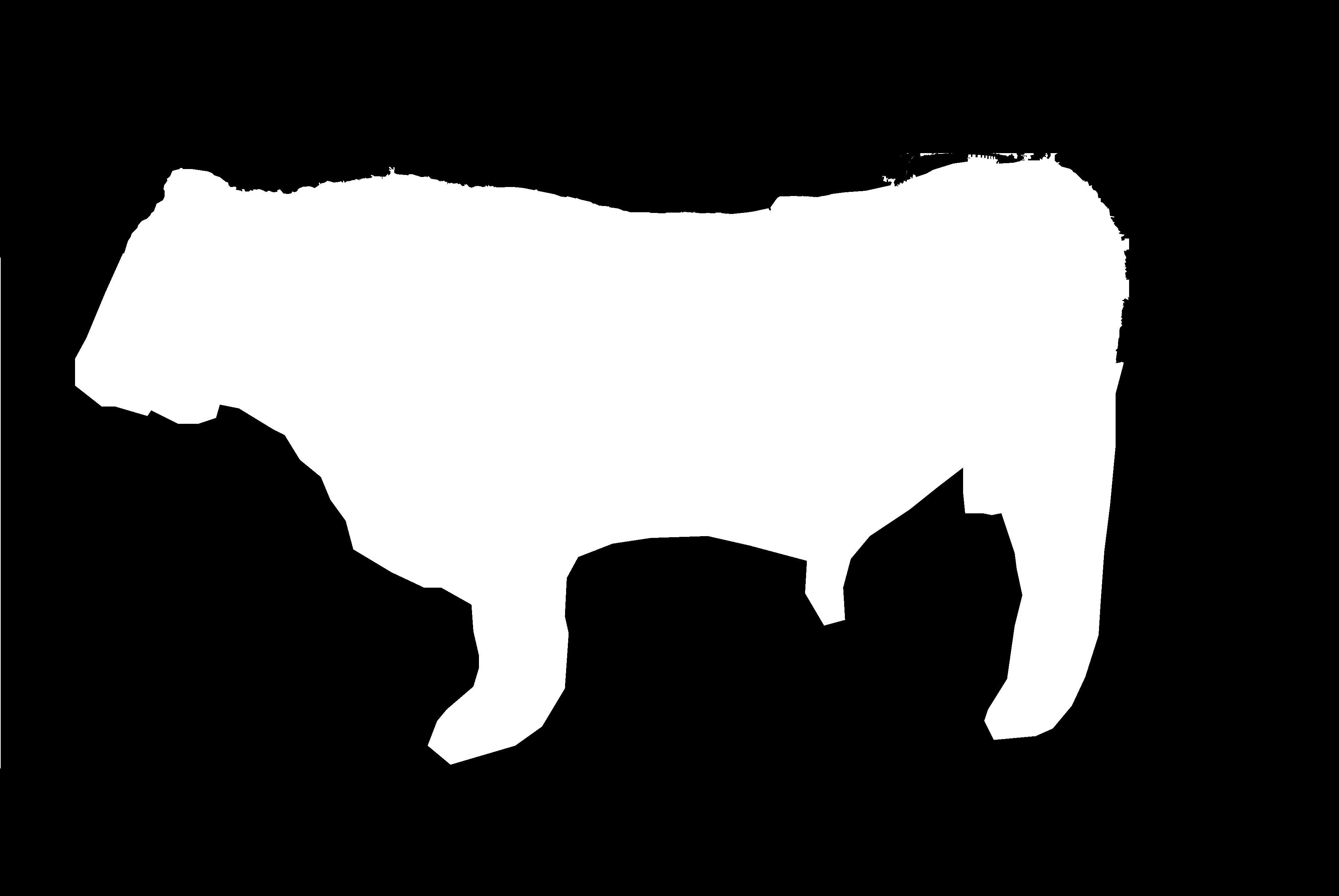

Supplement: Supplemental Information 3 — Different masks of the bull during model creation. [file peerj-cs-05-179-s003.zip › MASK per toro/DSC6146.png]

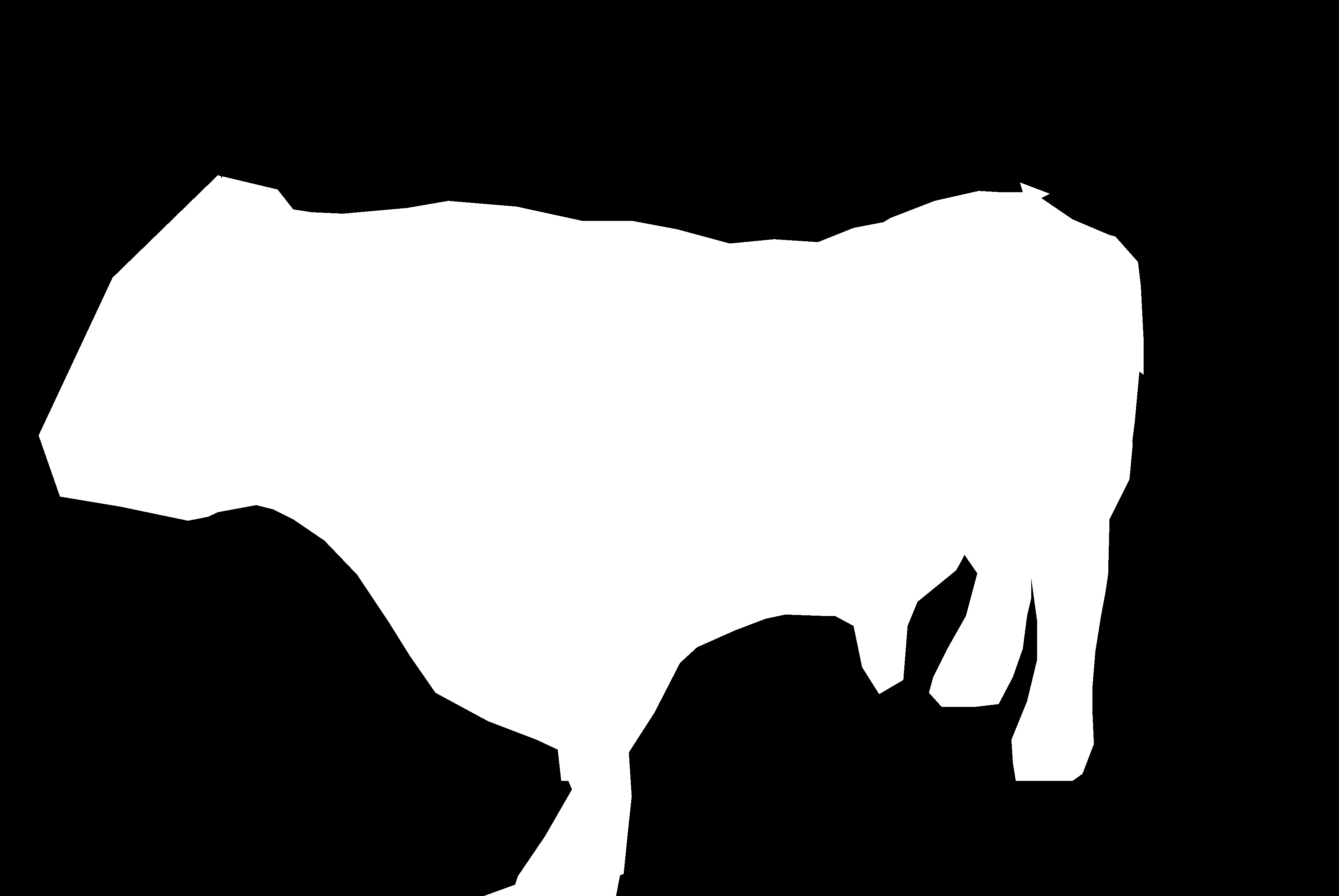

Supplement: Supplemental Information 3 — Different masks of the bull during model creation. [file peerj-cs-05-179-s003.zip › MASK per toro/DSC6165.png]

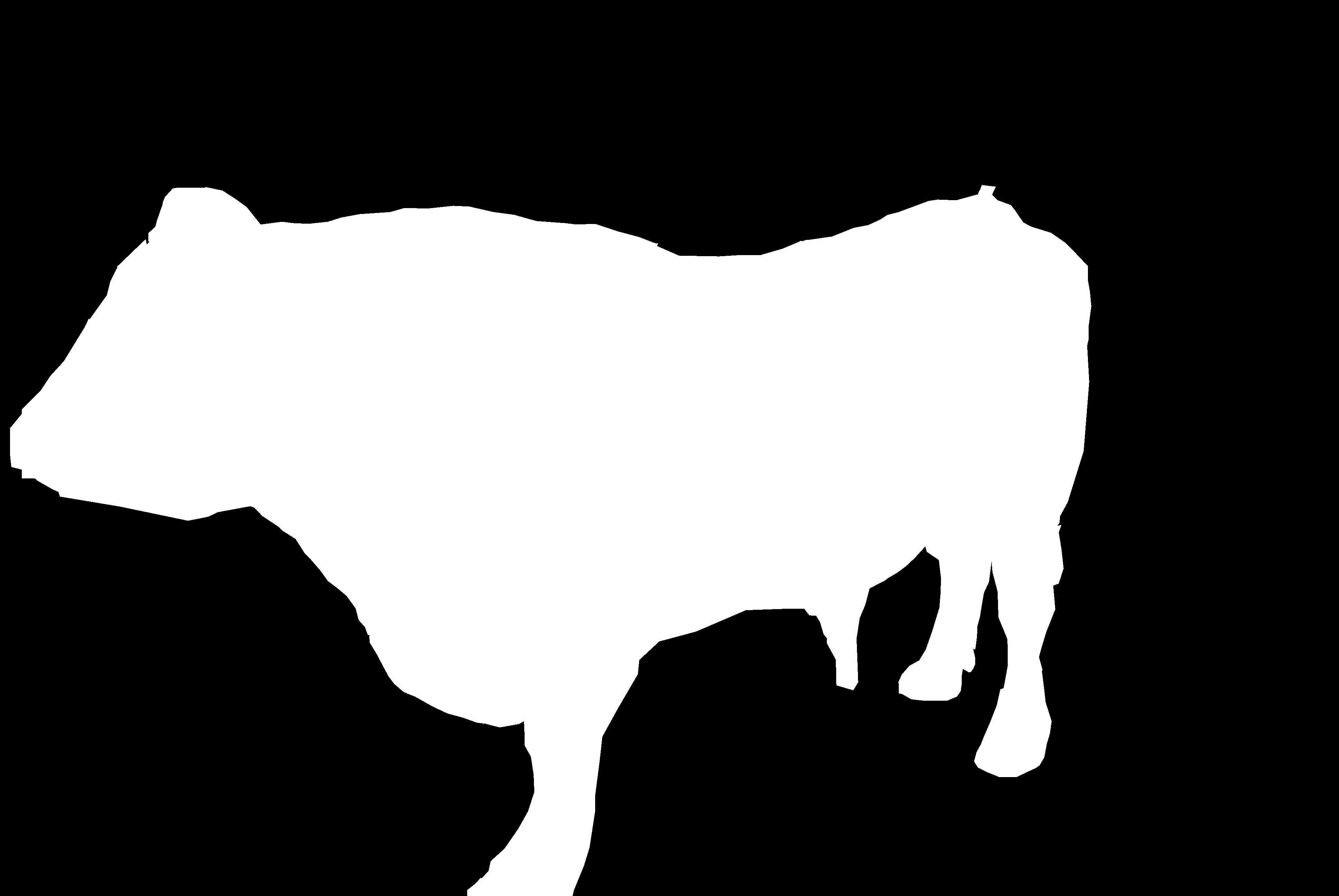

Supplement: Supplemental Information 3 — Different masks of the bull during model creation. [file peerj-cs-05-179-s003.zip › MASK per toro/DSC6167.png]

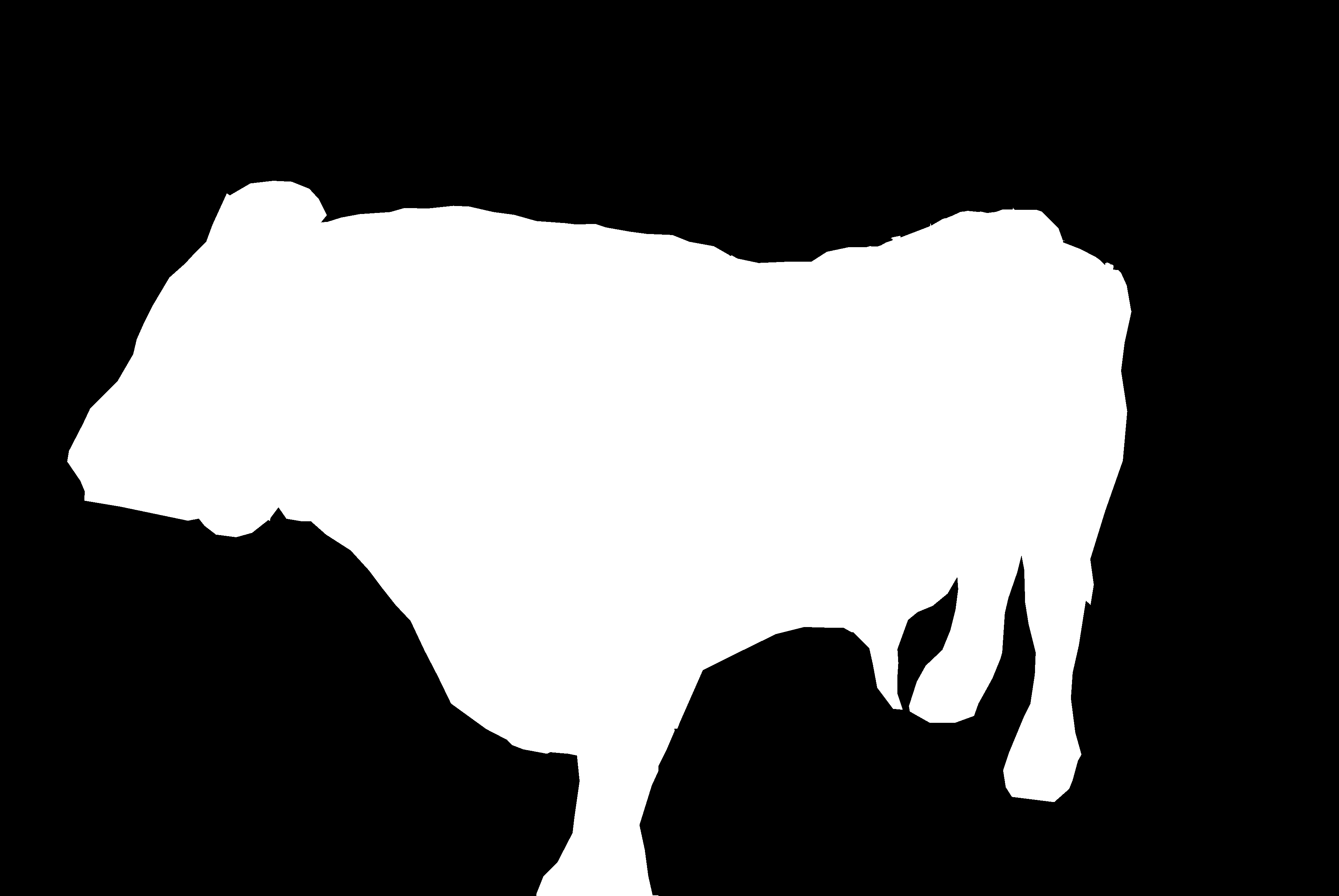

Supplement: Supplemental Information 3 — Different masks of the bull during model creation. [file peerj-cs-05-179-s003.zip › MASK per toro/DSC6168.png]

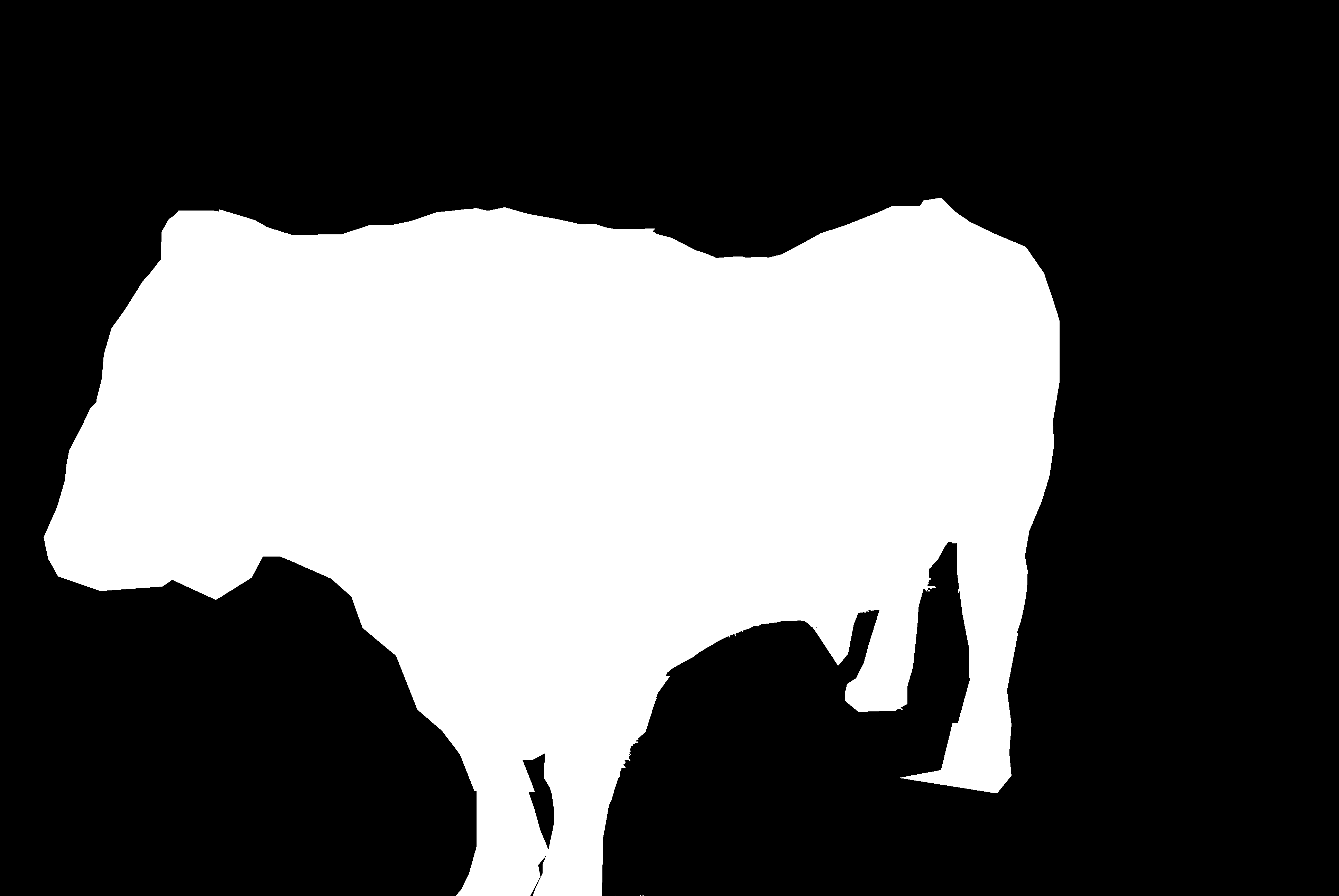

Supplement: Supplemental Information 3 — Different masks of the bull during model creation. [file peerj-cs-05-179-s003.zip › MASK per toro/DSC6171.png]

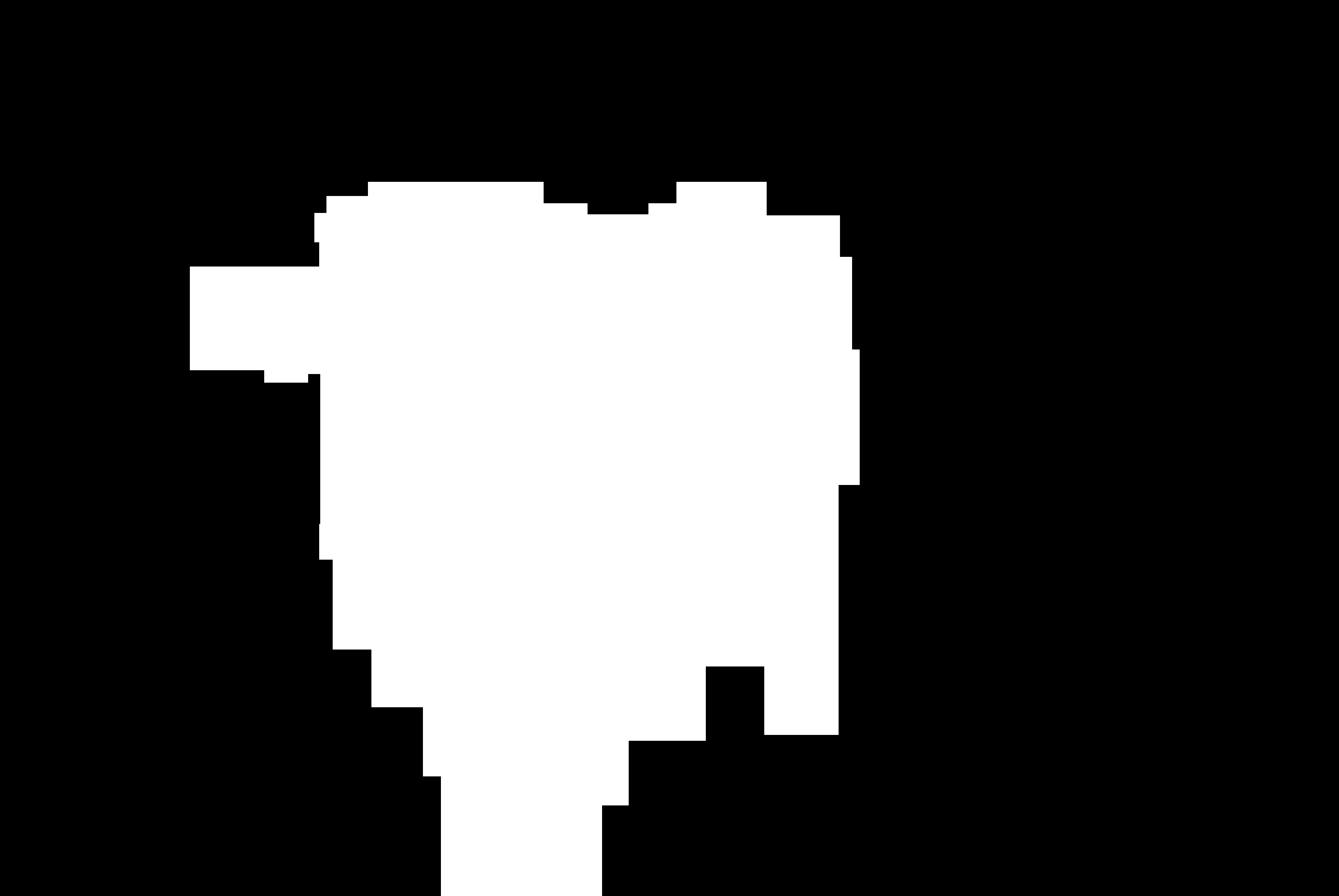

Supplement: Supplemental Information 3 — Different masks of the bull during model creation. [file peerj-cs-05-179-s003.zip › MASK per toro/DSC6177.png]

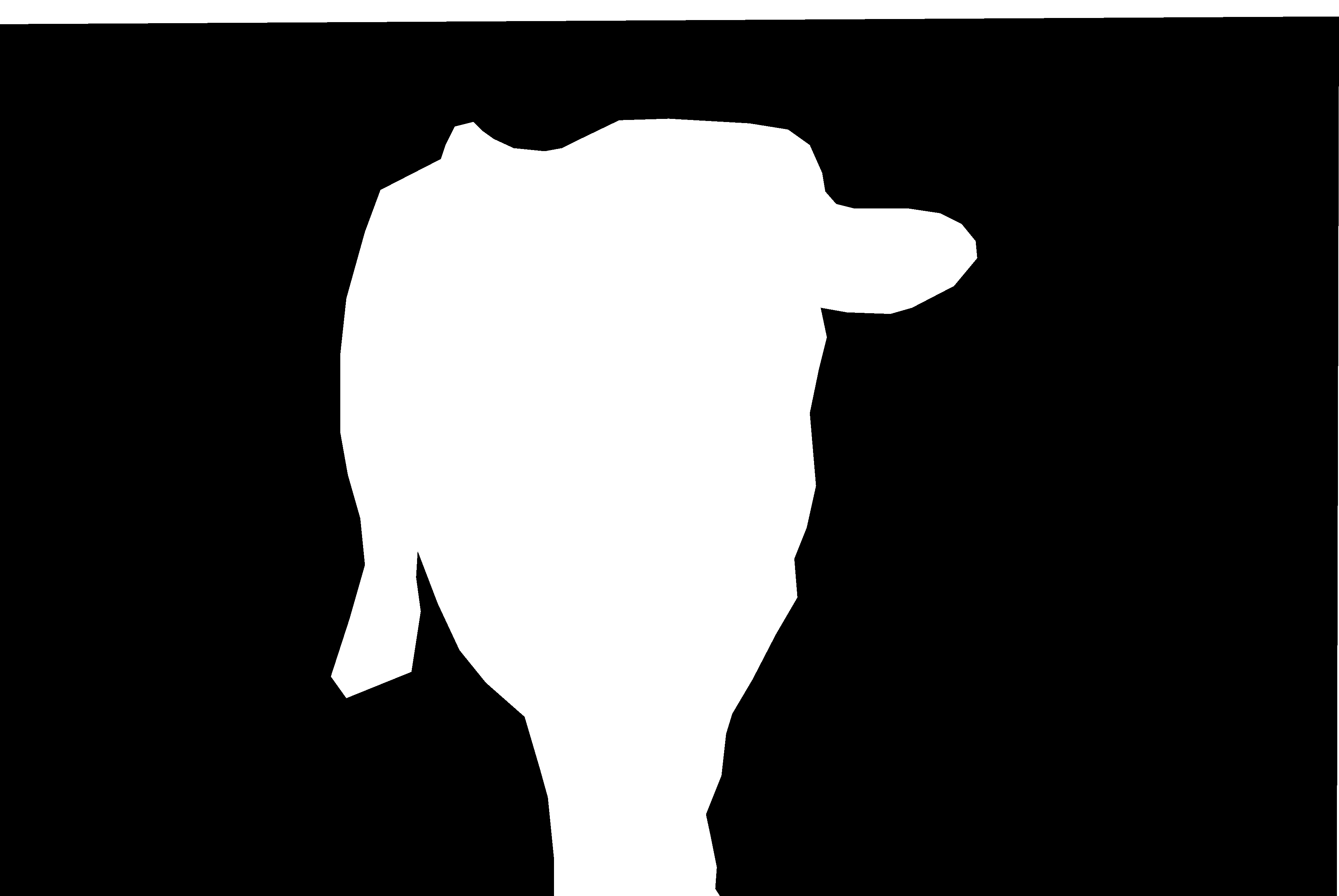

Supplement: Supplemental Information 3 — Different masks of the bull during model creation. [file peerj-cs-05-179-s003.zip › MASK per toro/dsc6189.png]

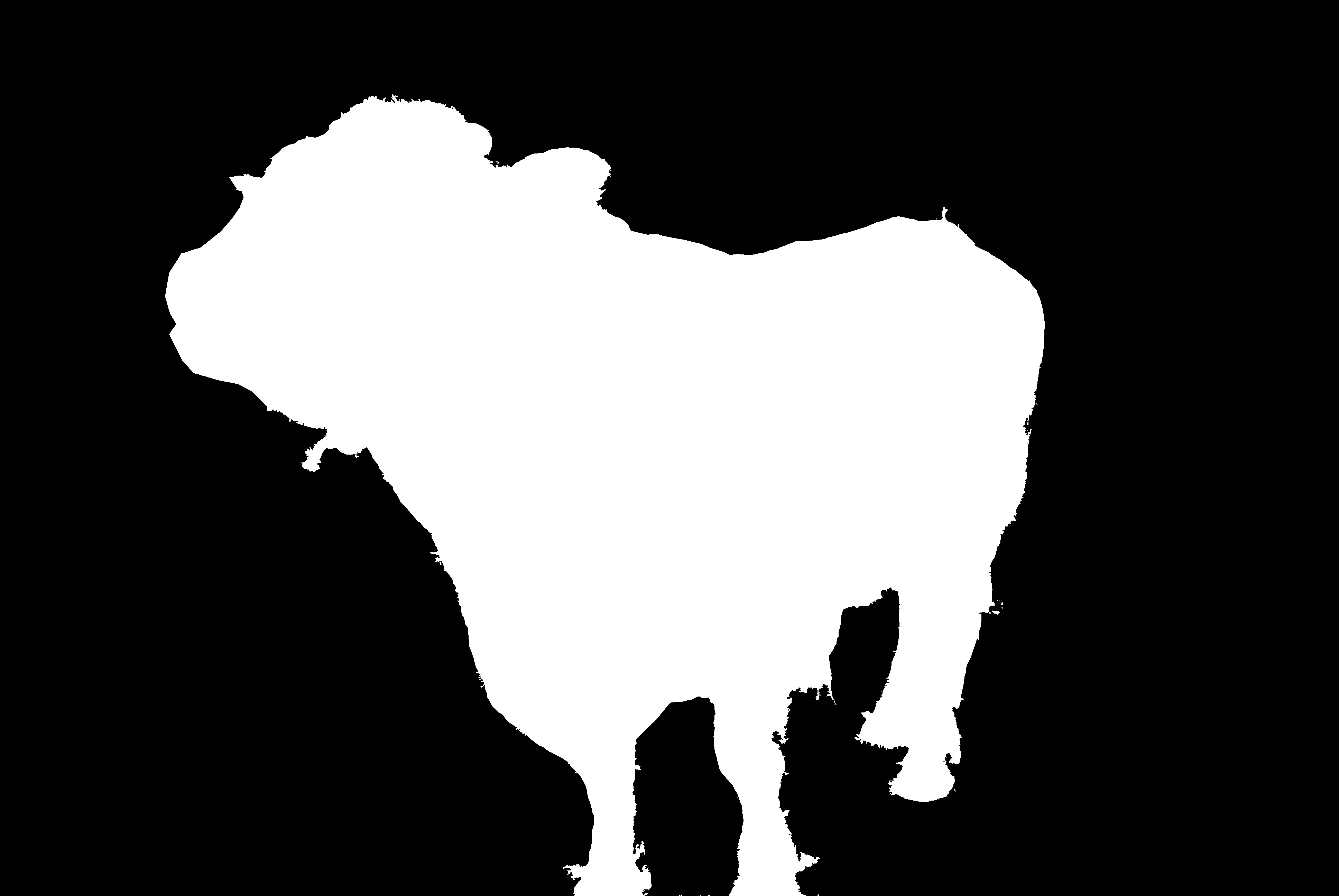

Supplement: Supplemental Information 3 — Different masks of the bull during model creation. [file peerj-cs-05-179-s003.zip › MASK per toro/DSC_6022.jpg]

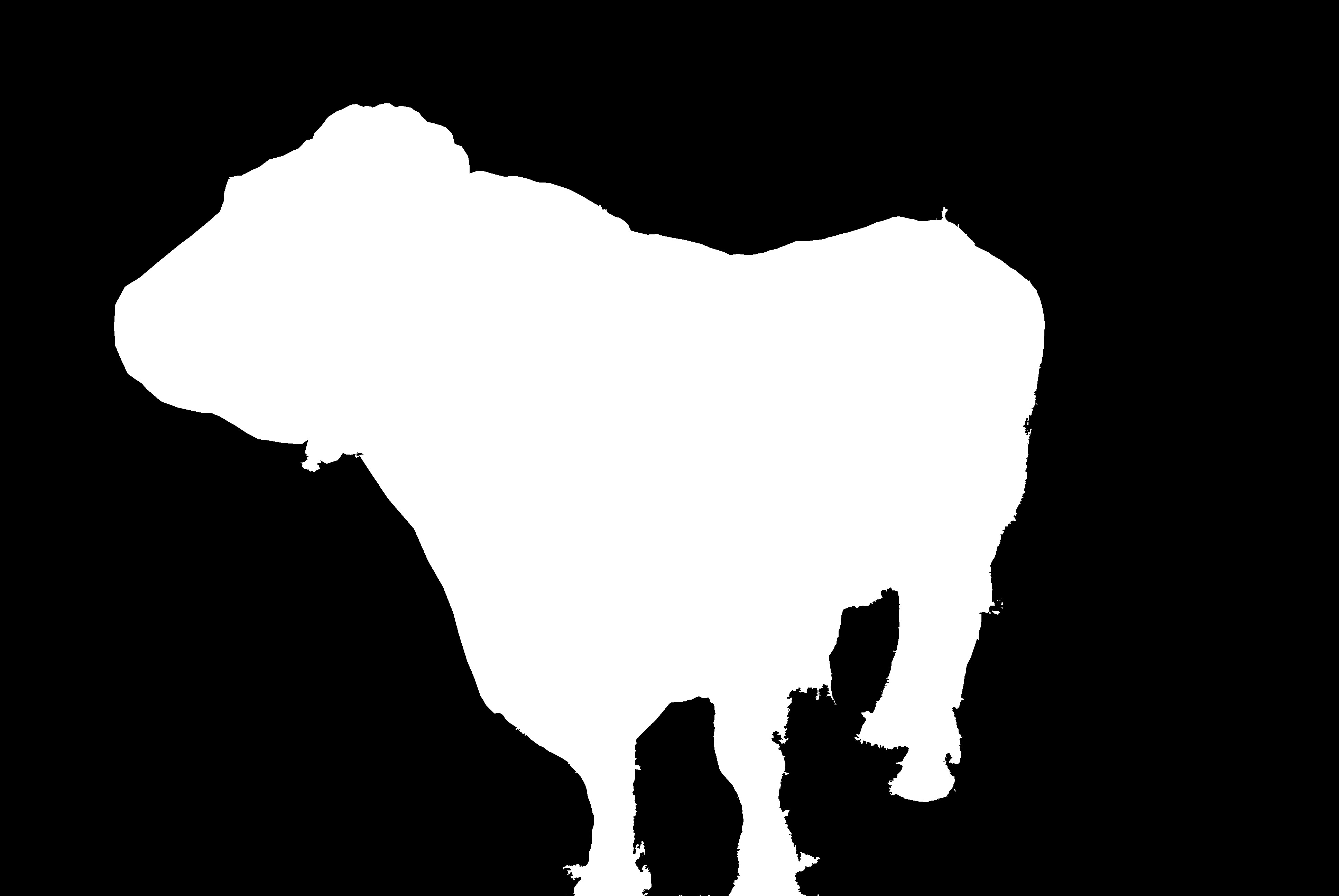

Supplement: Supplemental Information 3 — Different masks of the bull during model creation. [file peerj-cs-05-179-s003.zip › MASK per toro/DSC_6023.jpg]

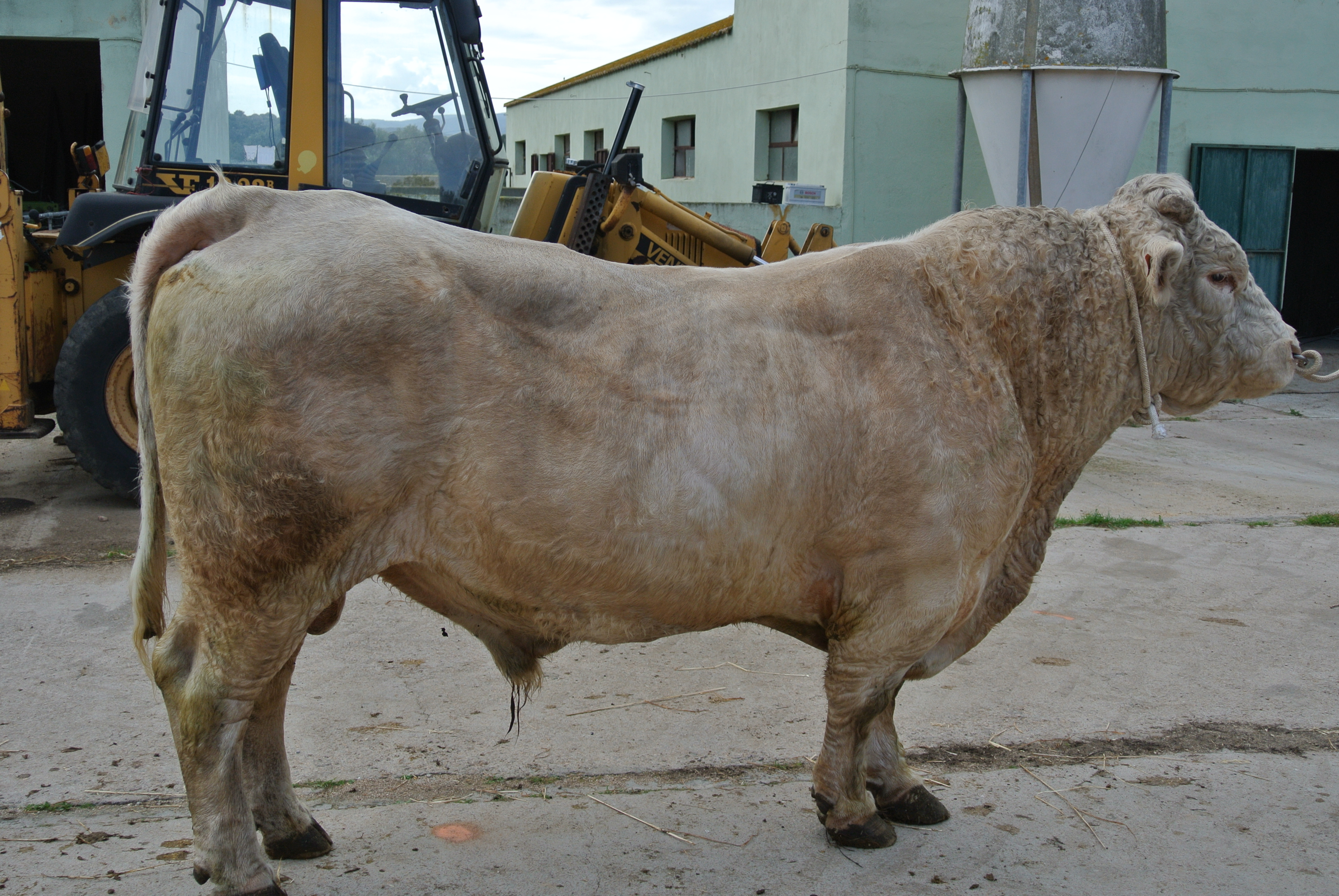

Supplement: Supplemental Information 3 — Different masks of the bull during model creation. [file peerj-cs-05-179-s003.zip › MASK per toro/DSC_6066.JPG]

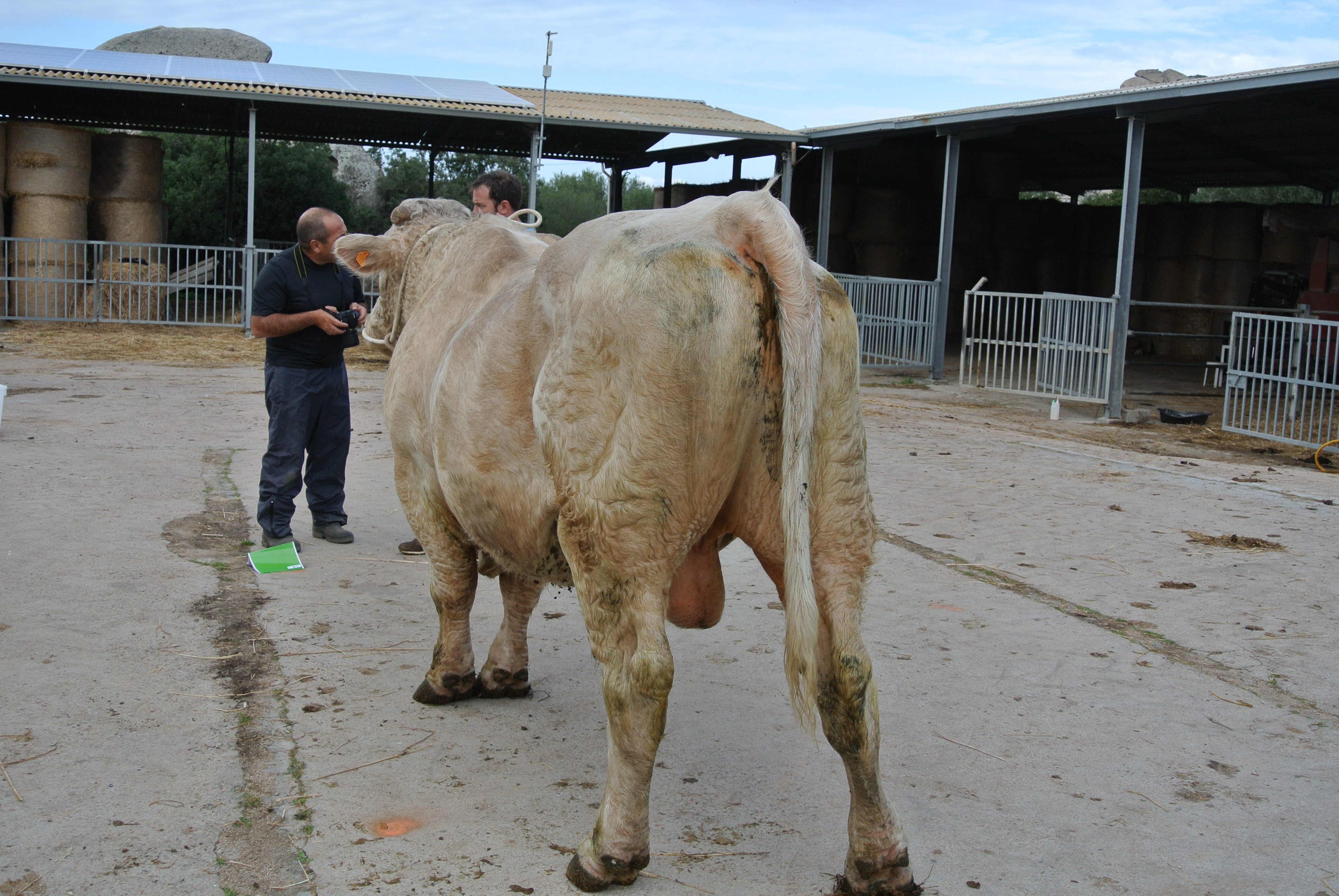

Supplement: Supplemental Information 3 — Different masks of the bull during model creation. [file peerj-cs-05-179-s003.zip › MASK per toro/DSC_6105.JPG]

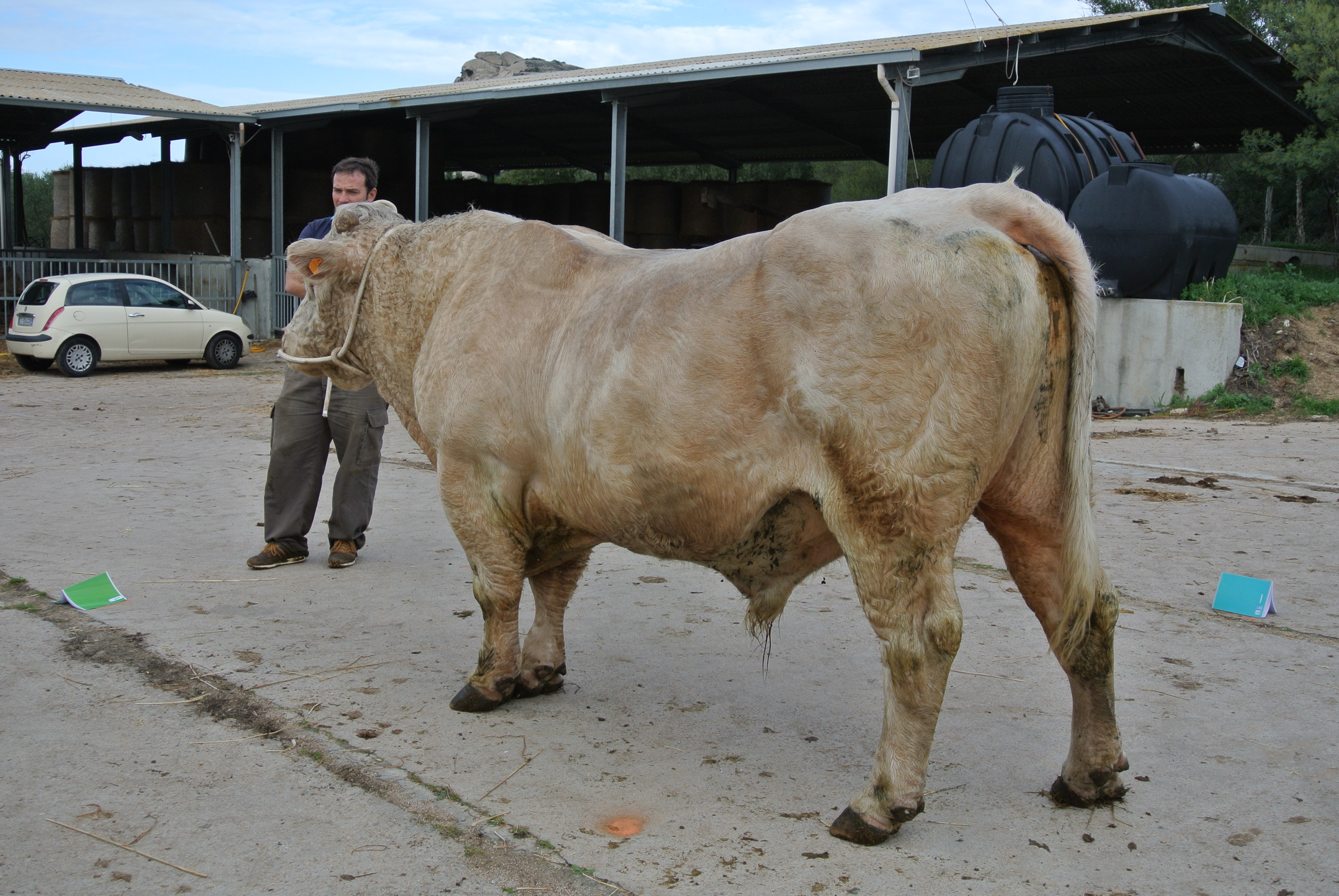

Supplement: Supplemental Information 3 — Different masks of the bull during model creation. [file peerj-cs-05-179-s003.zip › MASK per toro/DSC_6125.JPG]

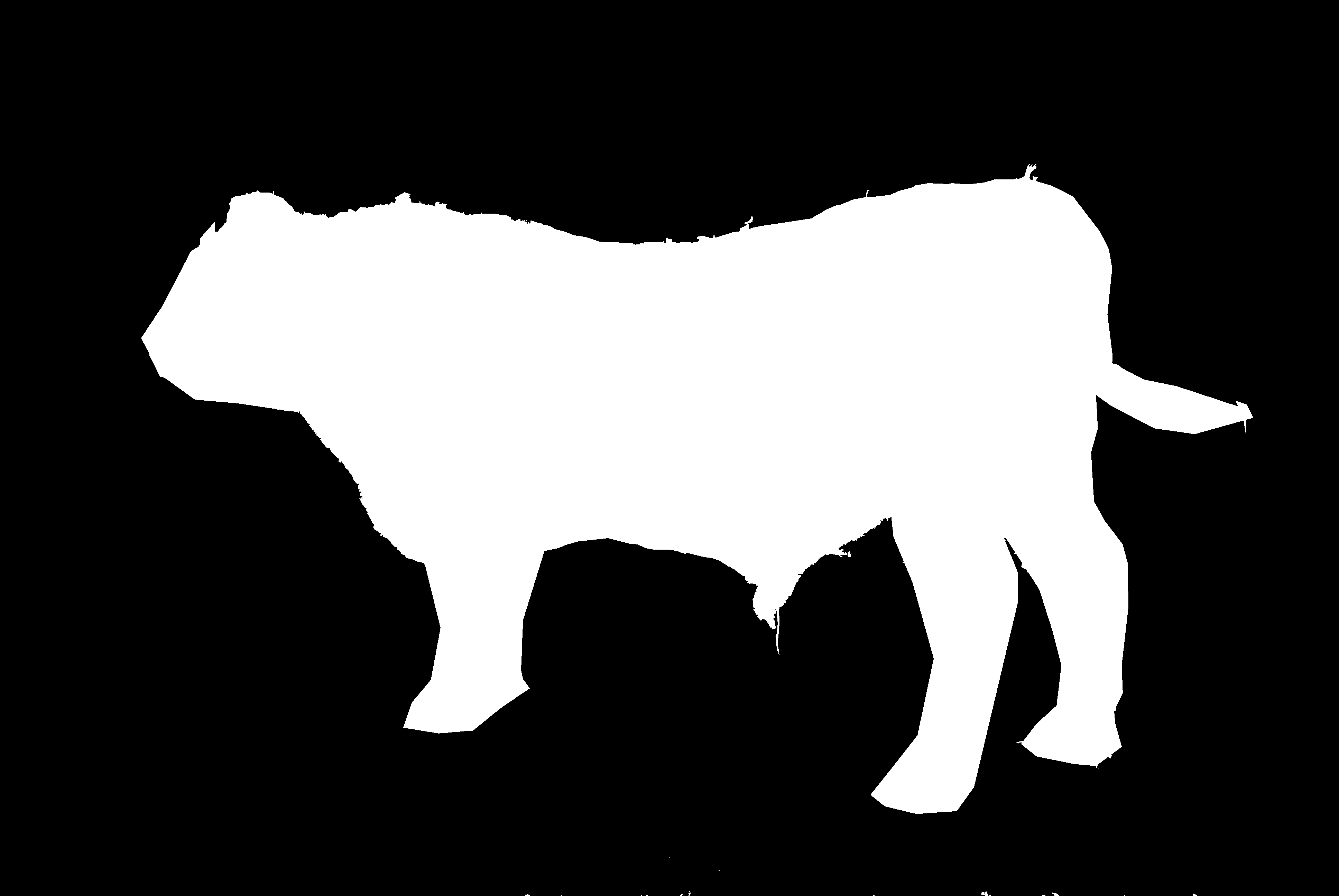

Supplement: Supplemental Information 3 — Different masks of the bull during model creation. [file peerj-cs-05-179-s003.zip › MASK per toro/toro.png]
